# Ultrasensitive and Self‐Powered Terahertz Detection Driven by Nodal‐Line Dirac Fermions and Van der Waals Architecture

**DOI:** 10.1002/advs.202102088

**Published:** 2021-10-19

**Authors:** Libo Zhang, Zhuo Dong, Lin Wang, Yibin Hu, Cheng Guo, Lei Guo, Yulu Chen, Li Han, Kaixuan Zhang, Shijian Tian, Chenyu Yao, Zhiqingzi Chen, Miao Cai, Mengjie Jiang, Huaizhong Xing, Xianbin Yu, Xiaoshuang Chen, Kai Zhang, Wei Lu

**Affiliations:** ^1^ Department of Optoelectronic Science and Engineering State Key Laboratory for Modification of Chemical Fibers and Polymer Materials Donghua University Shanghai 201620 China; ^2^ State Key Laboratory for Infrared Physics Shanghai Institute of Technical Physics Chinese Academy of Sciences 500 Yu‐tian Road Shanghai 200083 China; ^3^ CAS Key Laboratory of Nanophotonic Materials and Devices & Key Laboratory of Nanodevices and Applications i‐Lab Suzhou Institute of Nano‐Tech and Nano‐Bionics (SINANO) Chinese Academy of Sciences Ruoshui Road 398 Suzhou Jiangsu 215123 China; ^4^ School of Nano‐Tech and Nano‐Bionics University of Science and Technology of China Jinzhai Road 96 Hefei Anhui 230026 China; ^5^ Research Center for Intelligent Network Zhejiang Lab Hangzhou 311121 China; ^6^ School of Physics Southeast University Nanjing 211189 China; ^7^ The 50th Research Institute of China Electronics Technology Group Shanghai 200331 China; ^8^ School of Physical Science and Technology ShanghaiTech University Shanghai 201210 China

**Keywords:** nodal‐line semimetals, photo‐thermoelectric effect, terahertz photodetector, ZrGeSe single crystals

## Abstract

Terahertz detection has been highly sought to open a range of cutting‐edge applications in biomedical, high‐speed communications, astronomy, security screening, and military surveillance. Nonetheless, these ideal prospects are hindered by the difficulties in photodetection featuring self‐powered operation at room temperature. Here, this challenge is addressed for the first time by synthesizing the high‐quality ZrGeSe with extraordinary quantum properties of Dirac nodal‐line semimetal. Benefiting from its high mobility and gapless nature, a metal‐ZrGeSe‐metal photodetector with broken mirror symmetry allows for a high‐efficiency photoelectric conversion assisted by the photo‐thermoelectric effect. The designed architecture features ultrahigh sensitivity, excellent ambient stability, and an efficient rectified signal even above 0.26 THz. Maximum responsivity larger than 0.11 A W^−1^, response time of 8.3 µs, noise equivalent power (NEP) less than 0.15 nW Hz^−1/2^, and demonstrative imaging application are all achieved. The superb performances with a lower dark current and NEP less than 15 pW Hz^−1/2^ are validated through integrating the van der Waals heterostructure. These results open up an appealing perspective to explore the nontrivial topology of Dirac nodal‐line semimetal by devising the peculiar device geometry that allows for a novel roadmap to address targeted terahertz application requirements.

## Graphical abstract

Nodal‐line Dirac semimetals afford a suitable platform for low‐energy photodetection stemming from the novel quantum phenomena. Here, high‐speed THz detection is efficiently achieved through exploring light–matter interaction within nodal‐line Dirac fermions, and major improvement of performance has been envisioned by engineering novel architecture in combination with high‐quality nodal‐line ZrGeSe single‐crystal.

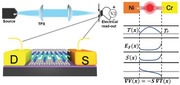

## 1 Introduction

The quest for novel terahertz (THz) detection featuring uncooled and self‐powered capabilities has received unprecedented research interest from scientific and technical communities, driven by its widespread applications in remote sensing, noninvasive imaging, wireless communication, and high‐resolution spectroscopy.^[^ ^1^, ^2^, ^3^, ^4^ ^]^ Commercially available THz detectors based on traditional semiconductors such as Golay cells, Schottky diodes, microbolometers, and others, suffer from severe drawbacks, failing to approach a good tradeoff between high‐speed, high‐sensitivity, and working temperature.^[^ ^5^ ^]^ A step‐change improvement could be supplied by exploring innovative materials and novel device architecture that invokes exceptional photodetection mechanisms.

The advent of 2D materials with peculiar structures and electronic properties provide versatile capabilities for designing flexible optoelectronic devices across a broad spectrum.^[^ ^6^, ^7^, ^8^ ^]^ Due to the Pauli blocking and quantum confinement effect, most 2D materials have congenital disadvantages for low‐energy photon detection due to the quantum‐confined large bandgap, weak optical absorption, and shorter carrier lifetime.^[^ ^9^ ^]^ The emergence of the topological semimetal (TSM) opens a door for developing self‐powered low‐energy photodetection, stemming from their gapless nature similar to graphene but with more intriguing physical properties.^[^ ^10^, ^11^, ^12^, ^13^, ^14^ ^]^ Benefiting from linear dispersion band structure protected by inversion‐symmetry, the detectable wavelength limited by bandgap is absent for TSM, and fast relaxation of nonequilibrium carriers enables high‐speed operation across a broad spectrum band.^[^ ^15^ ^]^ Since the first demonstration of TSM,^[^ ^16^ ^]^ a fruitful avenue of strategies has been proposed to develop innovative optical techniques by taking advantage of topologically nontrivial electronic band structures and quantum wavefunction behavior of the TSM, which will satisfy the massive needs for high‐frequency optoelectronics. Among such, 3D Dirac semimetal Cd_3_As_2_ has spawned highly sensitive photodetection with a simple configuration in the mid‐infrared region, and the graphene‐Cd_3_As_2_ hybrid system through layer‐by‐layer stacking exhibits the quantized conductance plateaus by local and nonlocal signals near the Dirac point.^[^ ^17^ ^]^ Additionally, due to the mid‐infrared bulk photovoltaic effect in the Weyl semimetal TaAs, the shift current separated from the photo‐thermoelectric effect is revealed via the polarization measurement.^[^ ^18^ ^]^ The shift current of Weyl semimetal TaIrTe_4_ is boosted up by the singularities of the Berry curvature at the vicinity of Weyl nodes, opening up novel feasibility to explore exotic photoresponse through the single‐particle excitation process in the mid‐infrared regime.^[^ ^19^ ^]^ However, these efforts toward energy harvesting at longer wavelengths are overwhelmed by the lack of a peculiar band structure that allows for addressing bottlenecks of the traditional single‐particle excitation.

Nodal‐line semimetals (NLS), which possess linearly dispersing bulk bands crossing points, creating a closed‐loop rather than discrete points in the Brillouin zone (BZ), encourage more newfangled fundamental researches and device applications for low‐energy photon detection due to novel quantum properties, including ultrahigh stability, quantized Hall effect, and colossal magnetoresistance.^[^ ^20^, ^21^, ^22^, ^23^ ^]^ Here, we demonstrate efficient THz detection by exploring light–matter interaction within nodal‐line Dirac fermions, where high‐quality material growth technology has implemented significant performance improvement in combination with novel device architecture.

## 2 Results

**Figure 1:**
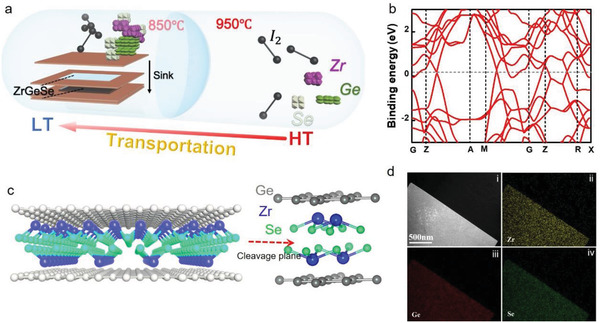
CVT synthesis and characterization of ZrGeSe single crystals. a) Schematic of the CVT growth method. b) Electronic band structure of the bulk ZrGeSe. c) The lattice structure of ZrGeSe. The natural cleavage plane gives rise to the neighboring Zr–Se layers, which possess relatively weak Zr–Se bonding. d) Element mapping. The scale bar corresponds to 500 nm.

The single crystal ZrGeSe is synthesized via the chemical vapor transport (CVT) method using the iodine (I_2_) as a transport agent and the high‐purity element powders of Zr, Ge, Se as the precursors in **Figure** **1**a (see the Experimental Section for details). Raman spectra, shown in Figure S1 (Supporting Information), is also performed out to confirm the chemical structure of multilayer exfoliated ZrGeSe flake, and three prominent Raman peaks B_1g_, A_2g_, and A_1g_ at 167, 179, and 222 cm^−1^ conform well with theoretically documented results.^[^ ^24^ ^]^ ZrGeSe characterization results (lattice parameter, lattice spacing, and multilayer thickness) in Figure S1 (Supporting Information) agree with density functional theory (DFT)‐calculated values and ZrGeSe is also proved as NLS with high mobility in Figure 1b. ZrGeSe has a PbClF‐type tetragonal crystal structure with P4/nmm space group,^[^ ^25^ ^]^ possessing a platelike stacking of Ge‐Zr‐Se‐Se‐Zr‐Ge slabs along the z‐axis in Figure 1c. Ge atoms alone form a square net located in the *x–y* plane, and Zr atoms are coordinated by four Se atoms. Figure 1d (i–iv) shows a low magnification scanning electron microscope (SEM) image and the uniform distribution of Zr, Ge, and Se elements by element mapping analysis.

**Figure 2:**
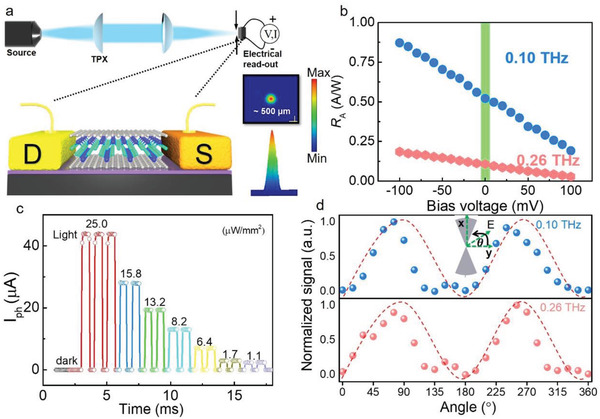
The performances of the ZrGeSe‐based photodetector. a) Schematic representation of measurement setup on the top. A THz spot with uniform intensity distribution measured by a terahertz camera is shown on the right. b) Bias‐dependence of detected signal at 0.26 THz, ranging from −0.1 to 0.1 V with 0.01 V step. c) Dynamic photosignal curve with different incident power densities. d) Polar diagram of photosignal characteristics as a function of *θ* (*θ* = 90^°^: fan‐shaped antenna axis parallel to the radiation polarization), inset shows an optical image of the device and the photoactive region of ZrGeSe channel.

The ZrGeSe‐based device is designed in a planar structure contacted by fan‐shaped electrodes (source (S) and drain (D) electrodes) with dissimilar metals in **Figure** **2**a. The source and drain electrodes with the 8 µm channel length are materialized with electron beam lithography combined with the evaporation of Cr/Au and Ni/Au metallic stacks. In this structure, an asymmetry is introduced that enables the generation of THz detection signal stemming from different Seebeck coefficients of Cr and Ni contacts. The corresponding optical and atomic force microscopy (AFM) images are presented in Figure S3 (Supporting Information). THz radiation is generated from a VDI multiplier‐link and collimated by two polymethylpentene (TPX) lenses with focal lengths of 100 mm (Measurement setup in Figure 2a and details see Experimental Section). Meanwhile, the photoresponse measurements are performed by using a lock‐in technique. The linear current–voltage (*I*–*V*) curve in the dark shows an excellent ohmic contact between the material and metal stacks (Figure S3, Supporting Information). When the THz beam at normal incidence impinges onto the channel, the antenna near the channel will accumulate induction oscillating charges and yields the surface carriers oscillation in ZrGeSe, during which the electromagnetic wave is converted effectively into a direct current. The selective frequency‐dependence of photoresponse from 0.072–0.12, 0.23–0.30 THz is shown in Figure S3 (Supporting Information) due to the absorption of the THz photon at different frequencies and the antenna geometry. By following the formula of *R*_A_ = *I*_ph_/*PS*_*λ*_, the derived peak responsivity can reach 0.56 A W^−1^ at 0.10 THz and 0.11 A W^−1^ at 0.26 THz, respectively. By applying a bias voltage across the S–D electrodes (the S electrode is grounded), there is a significant photocurrent change contributed by the photocarriers that are accelerated along with the electric field (Figure 2b). To further validate the performance of the ZrGeSe photodetector in the THz region, dynamic photosignal curves with various incident power densities are measured at ambient temperature without a bias voltage (Figure 2c). The linear dynamic range as an important property for a photodetector can be quantified by measuring photocurrent (*I*_ph_) under different power intensities (Details see the Experimental Section and Figure S4 in the Supporting Information), and excellent linearity (36 dB) validates the square‐law of electric‐field dependent photoresponse. The polarization dependence of our device is also measured via changing of beam polarization‐angle *θ*, as shown in Figure 2d, in which photoresponse is peaked when the fan‐shaped antenna axis is in line with the polarization of radiation. In the meantime, the polarization angle corresponding to the maximum photoresponse remains the same at different incident frequencies.

**Figure 3:**
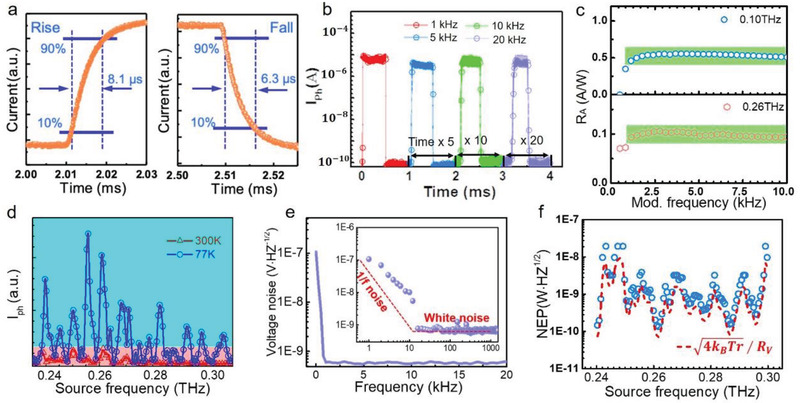
Optical characterization. a) Time‐resolved photosignal of ZrGeSe‐based photodetector at *V*_ds_ = 0 V. b) The pulse response measurement at the electronic modulated frequency of 1, 5, 10, and 20 kHz under ambient conditions. c) Responsivity as a function of the modulation frequency at 0.1 and 0.26 THz. d) Frequency dependence of the photosignal of the device, measured at *V*_ds_ = 0 V. e) Voltage noise spectra of the ZrGeSe device without external bias is a reference for the 1/*f* noise trend. The fitted red line is a reference for the 1/*f* noise trend. f) Optical noise equivalent power (NEP) extracted as the ratio between the thermal Johnson–Nyquist noise spectral density and responsivity (*R*_V_) (red dotted line), and as the ratio between the measured noise voltage (*N*_V_) and responsivity (*R*_V_) (blue solid ball).

To further characterize the device performance, the response time, routinely defined as the time required by the photoresponse rising from 10% to 90% or falling from 90% to 10% for a single impulse, is retrieved directly from the high‐speed sampling oscilloscope. **Figure** **3**a displays the rise time of ≈8.1 µs and the fall time of ≈6.3 µs at electrically modulated frequencies of over 1 kHz, attributed to more efficient carriers collection with the intraband process. The pulsed photosignals at the electrically modulated frequency of 1, 5, 10, and 20 kHz are comparatively displayed in Figure 3b, showing no noticeable photocurrent deterioration. The modulation frequency‐dependence of the responsivity at 0.10 and 0.26 THz in Figure 3c substantiates both the high‐sensitivity and high‐speed capabilities. By further decreasing the operation temperature, the photocurrent of our device exhibits a significant improvement at 77 K (Figure 3d), which is attributable to the transport improvement of the material, and the resonance frequency remains rigidly unchanged, as is determined mainly by the antenna structure and material absorption‐ability. To further assess the photodetection sensitivity, noise equivalent power (NEP) is calculated as the ratio between noise voltage density and responsivity (*v*_n_ */R*). Noise spectral density (Figure 3e) reflects differently dominant noise sources in the whole system, including 1/*f* noise, shot noise, and Johnson–Nyquist noise (thermal noise, *v*_t_). The 1/*f* noise originates from the changing of electronic states, prevailing at low frequencies (below 1 kHz). The shot noise is caused by the random generation of photogenerated carriers under radiation or thermal excitation of the detector. The thermal noise is associated with the ohmic resistance and temperature of the detector, generated by the random thermal motion of charge carriers. In this system, 1/*f* noise can be negligible since our device remains highly efficient even at the modulated frequency over 1 kHz,^[^ ^26^, ^27^ ^]^ and operating in zero‐bias condition, the contribution of shot noise to the entire noise spectrum can also be ignored.^[^ ^28^ ^]^ Thermal noise *v*_t_ contribution to noise spectral density via *v*_t_ =$\sqrt{4k_{B}Tr}\;$ is ≈3.95 nV Hz^−1/2^ at room temperature, is in the same order of magnitude as the results we measured experimentally (Figure 3e). As an essential performance metric, optical NEP, including the contribution of the antennas and the measurement circuit, can be approximated as *v*_t_/*R*_v_, and the minimum value of NEP is around 150 pW H_Z_^−1/2^ at ambient temperature (Figure 3f).

**Figure 4:**
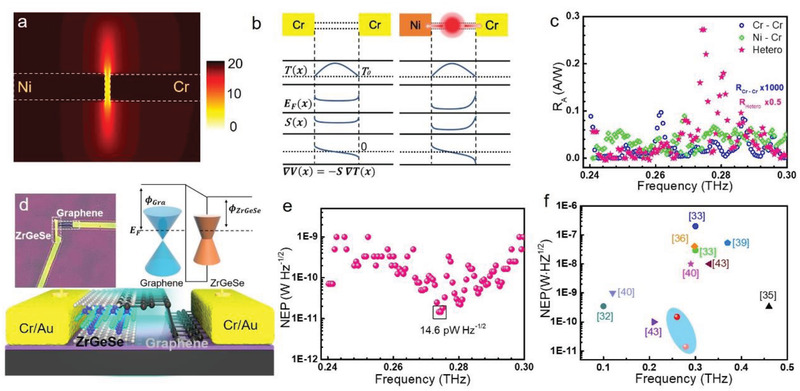
a) Simulated THz enhancement field distribution based on Ni–Cr electrodes and b) Profiles across the carrier temperature *T*(*x*), Fermi energy *E*_F_(*x*), Seebeck coefficient *S*(*x*), and potential gradient based on Cr–Cr electrodes without bias voltage and Ni–Cr electrodes without a bias voltage. c) The frequency dependence of the responsively for the device based on similar, dissimilar metal contact and heterojunction, respectively. d) Schematic representation of the ZrGeSe‐graphene vdW heterostructure device. Inset: Optical image of the heterostructure‐based device and schematic band diagram of ZrGeSe‐graphene heterostructure without bias voltage. E_F_ and ϕ denote the Fermi level and work function, respectively. e) NEP as a function of the frequency without a bias voltage. The minimum value of NEP is around 14.6 pW HZ^−1/2^. f) The comparison of reported typical 2D materials THz photodetectors at 300K (data taken from Refs.[32, 33, 34, 35, 36, 37, 38, 39, 40, 41, 42, 43, 44].

By adopting symmetric contact‐patterning, the photodetector exhibits negligence photoresponse. Instead, an asymmetric configuration with dissimilar metallic contact leads to directional thermoelectric detection. Meanwhile, the finite difference time domain (FDTD) simulation is also implemented to investigate the effect of different metal contact schemes. The localized field distribution in the channel in **Figure** **4**a indicates that the asymmetric antenna structure could focus THz photons more effectively than similar metal contacts. There is clear evidence based on experimental results that obey our foregoing inference by comparing the performance between two device configurations (Figure 4c). Here, a simple analytical model is presented to delineate the underlying physics process of photocurrent generation in Figure 4b.^[^ ^29^ ^]^ The locally heated carriers under THz radiation and rapidly heat transferring by metallic pads produce a heat‐driven carrier flowing as a result of gradient temperature‐distribution near the photoactive region. Owing to the dissimilar Seebeck coefficients *S*(*E*_f_) between the source and drain metal contacts, the diffusion of hot carriers induces a potential gradient ∇*V* (*x*)= ‐Δ*S*(*E*_f_)∇*T*(*x*), and the photo‐thermoelectric (PTE) current is given by *I*_PTE_ $\propto \;{\left|E\right|}^{2}\int \nabla V\left(x\right)$, where |*E*|^2^ is the electric field distribution.^[^ ^30^, ^31^ ^]^ We find that ZrGeSe could be a suitable candidate for engineering THz detection, and photosignal can be flexibly manipulated without bias voltage and with the advantages of low thermal noise and power consumption.

An attractive and reasonable strategy to further suppress the dark noise is to integrate ZrGeSe with the few‐layer graphene, which is immune to lattice mismatch via van der Waals (vdW) stacking shown in Figure 4d (inset: the optical image of ZrGeSe/graphene heterostructure). Upon THz illumination, the different light‐absorption abilities of ZrGeSe or graphene layers can give rise to a temperature gradient at the interface of the stacked region and intraband transport of nonequilibrium carriers across the heterostructure can be activated. These carriers would directionally flow owing to different band alignments between two materials in the inset of Figure 4d and are collected by the electrode, leading to the pronounced photoresponse even at 0.27 THz. Through thermal‐agitation noise‐suppressing, this vdW heterostructure improves detection sensitivity apparently, and NEP ≈14.6 pW Hz^−1/2^ is achieved under the 0.27 THz illumination Figure 4e (more details see Figure S5 in the Supporting Information), which is much lower than most reported room‐temperature THz photodetectors based on typical 2D materials in Figure 4f. Thus, the room‐temperature photodetection capability of the ZrGeSe‐graphene vdW photodetector with low dark current and high sensitivity opens a unique path to extensive area application in portable optoelectronic devices.

**Figure 5:**
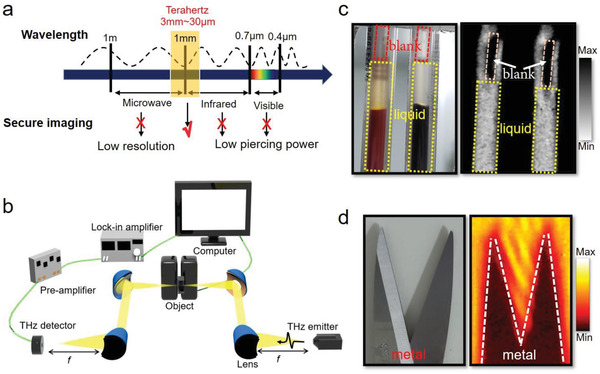
THz imaging. a) Security imaging application of the ZrGeSe‐based photodetector in the electromagnetic spectra. b) Scheme diagram of the experimental setup for THz imaging. c,d) Photographs of the refill and metallic scissor and their raster scanning imaging at 0.26 THz. The objects are revealed in an envelope, which is invisible to the naked eye.

Typically, visible/infrared imaging can hardly penetrate many targeted objects, and the low resolution of microwave‐based imaging hampers the observable details. In this regard, THz imaging will be an excellent candidate to possess the unique advantages of both imaging routes with the aim of suitable resolution and penetrability (**Figure** **5**a). Our ZrGeSe photodetector with fast response, remarkable NEP, and stability has already been exploitable for practical application in a series of large‐area transmission‐imaging verification. The THz beam is focused by two pairs of off‐axis parabolic mirrors, and the images are acquired by raster‐scanning the target objects at the beam focus, consisting of the 200 × 200‐pixel image with a 20 ms integration time at pixel (Figure 5b). Transmission images for ink and metallic scissor inside an envelope are revealed in Figure 5c,d. Even though objects are enclosed and invisible to the human eyes, the details of target objects could be seen by the ZrGeSe photodetector.

## 3 Conclusions

Here, high‐quality single‐crystal growth and device implementation of a topological nodal‐line semimetal ZrGeSe have been reported for the first time. The THz photodetector with ultrahigh sensitivity, low NEP, good air‐stability has been developed by utilizing the superior dynamics of nodal‐line Dirac fermions at room temperature. The responsivity being larger than 0.11 A W^−1^ of ZrGeSe‐based photodetector with a simple configuration at the self‐powered mode is superior to any other 2D material‐based detectors operating at room temperature by utilization of the photo‐thermoelectric effect. Unlike conventional THz detectors hampered by the lack of tradeoff between sensitivity, frequency, and speed, our work provides a pioneering solution for circumventing this difficulty via quantum engineering, where the novel quantum materials implemented in an exceptionally designed geometry offer a unique advantage in THz detection beyond drawbacks imposed by band‐structure engineering. Our results open up a fruitful avenue for research of nontrivial topological phenomena in the field of THz photonics, which could meet the urgent requirements for next‐generation communication and intelligent sensing systems.

## 4 Experimental Section

### Single‐Crystal Growth

High‐quality ZrGeSe single crystals were grown by the chemical vapor transport (CVT) method using iodine (*I*_2_) as a transport agent. The intimate mixture of high‐purity element powder of Zr, Ge, Se (≈1.5 g, 200 meshes), together with 10 mg mL^−1^ iodine, was enclosed in evacuated silica glass tubes as the precursor for the growth. Then the silica glass tubes were placed horizontally in the two‐zone quartz tube furnace between 950 °C (hot zone, source materials) and 850 °C (cold zone, sink) for one week. The ZrGeSe single crystals with a typical size of 2 × 2 mm^2^ and shiny surfaces can be found at the cold end of the silica glass tubes.

### Photocurrent Measurements

THz frequency was tuned up to 0.26 THz (WR 2.8 Tripler) and 0.10 THz (WR 9 Tripler) from the VDI multipliers connected to a 40‐GHz microwave source. The THz output beam was collimated by a set of two polymethylpentene (TPX) lenses, resulting in a 500 µm diameter focal beam spot while its amplitude was modulated as a square wave at 1.1 kHz. The power output was calibrated by a TK100 power meter. The entire measurement system was built based on a closed‐circuit configuration through the lock‐in amplifier technique. The responsivity *R*_A_ was retrieved from *I*_ph_ via the relation *R*_A_ = *I*_ph_/*PS*_*λ*_, where *P* is the output power intensity, and *S*_*λ*_ is the active detection area (*S*_*λ*_ = *λ* ^2^/4*π*). For NEP analysis, the voltage noise spectra were measured using amplifiers and then digitized with a dynamic signal analyzer‐SR785. The optical NEP is evaluated from the responsivity of the device based on incident power, rather than absorbed power, which is experimentally and theoretically evaluated as *v*_n_/*R*_v_, where *v*_n_ is the root mean square of the noise voltage and *R*_v_ is the voltage responsivity: *R*_v_ = *r* × *R*_A_, *r* is device resistance. To provide the lower limit of noise figure, the thermal noise *v*_t_ was extracted from the electrical characteristic of the device via *v*_n_ = *v*_t_ = (4*k*_B_ *Tr*)^1/2^, where *k*_B_ is Boltzmann constant, *T* is temperature.

## Conflict of Interest

The authors declare no conflict of interest.

## Supporting information

Supporting Information

## Data Availability

The data that support the findings of this study are available from the corresponding author upon reasonable request.

## References

[1] D. Suzuki , S. Oda , Y. Kawano , Nat. Photonics 2016, 10, 809.

[2] F. Sizov , Semicond. Sci. Technol. 2018, 33, 123001.

[3] Philippe Tassin , Thomas Koschny , C. M. Soukoulis , Science 2013, 341, 620. 23929971 10.1126/science.1242253

[4] V. Pacheco‐Peña , Electronics 2021, 10, 268.

[5] I. Mehdi , J. V. Siles , C. Lee , E. Schlecht , Proc. IEEE 2017, 105, 990.

[6] L. Li , Y. Yu , G. J. Ye , Q. Ge , X. Ou , H. Wu , D. Feng , X. H. Chen , Y. Zhang , Nat. Nanotechnol. 2014, 9, 372. 24584274 10.1038/nnano.2014.35

[7] A. Tredicucci , M. S. Vitiello , IEEE J. Sel. Top. Quantum Electron. 2014, 20, 130.

[8] Zongyou Yin , Hai Li , Hong Li , Lin Jiang , Yumeng Shi , Yinghui Sun , Gang Lu , Qing Zhang , Xiaodong Chen , H. Zhang , ACS Nano 2012, 6, 74. 22165908 10.1021/nn2024557

[9] X. Liu , Q. Guo , J. Qiu , Adv. Mater. 2017, 29, 1605886. 10.1002/adma.201605886 28225160

[10] A. Mosca Conte , O. Pulci , F. Bechstedt , Sci. Rep. 2017, 7, 45500. 28383018 10.1038/srep45500 PMC5382546

[11] K. Kang , T. Li , E. Sohn , J. Shan , K. F. Mak , Nat. Mater. 2019, 18, 324. 30804510 10.1038/s41563-019-0294-7

[12] C. Zhang , Z. Ni , J. Zhang , X. Yuan , Y. Liu , Y. Zou , Z. Liao , Y. Du , A. Narayan , H. Zhang , T. Gu , X. Zhu , L. Pi , S. Sanvito , X. Han , J. Zou , Y. Shi , X. Wan , S. Y. Savrasov , F. Xiu , Nat. Mater. 2019, 18, 482. 30886399 10.1038/s41563-019-0320-9

[13] L. Zhang , Z. Chen , K. Zhang , L. Wang , H. Xu , L. Han , W. Guo , Y. Yang , C. N. Kuo , C. S. Lue , D. Mondal , J. Fuji , I. Vobornik , B. Ghosh , A. Agarwal , H. Xing , X. Chen , A. Politano , W. Lu , Nat. Commun. 2021, 12, 1584. 33707448 10.1038/s41467-021-21906-w PMC7952558

[14] C. Guo , Y. Hu , G. Chen , D. Wei , L. Zhang , Z. Chen , W. Guo , H. Xu , C.‐N. Kuo , C. S. Lue , X. Bo , X. Wan , L. Wang , A. Politano , X. Chen , W. Lu , Sci. Adv. 2020, 6, eabb6500. 32917593 10.1126/sciadv.abb6500 PMC7467699

[15] Q. Wang , J. Zheng , Y. He , J. Cao , X. Liu , M. Wang , J. Ma , J. Lai , H. Lu , S. Jia , D. Yan , Y. Shi , J. Duan , J. Han , W. Xiao , J. H. Chen , K. Sun , Y. Yao , D. Sun , Nat. Commun. 2019, 10, 5736. 31844067 10.1038/s41467-019-13713-1 PMC6915719

[16] A. A. Burkov , Nat. Mater. 2016, 15, 1145. 27777403 10.1038/nmat4788

[17] Y. F. Wu , L. Zhang , C. Z. Li , Z. S. Zhang , S. Liu , Z. M. Liao , D. Yu , Adv. Mater. 2018, 30, 1707547. 10.1002/adma.201707547 29995347

[18] G. B. Osterhoudt , L. K. Diebel , M. J. Gray , X. Yang , J. Stanco , X. Huang , B. Shen , N. Ni , P. J. W. Moll , Y. Ran , K. S. Burch , Nat. Mater. 2019, 18, 471. 30833781 10.1038/s41563-019-0297-4

[19] J. Ma , Q. Gu , Y. Liu , J. Lai , P. Yu , X. Zhuo , Z. Liu , J. H. Chen , J. Feng , D. Sun , Nat. Mater. 2019, 18, 476. 30833780 10.1038/s41563-019-0296-5

[20] J. Hu , Z. Tang , J. Liu , X. Liu , Y. Zhu , D. Graf , K. Myhro , S. Tran , C. N. Lau , J. Wei , Z. Mao , Phys. Rev. Lett. 2016, 117, 016602. 27419579 10.1103/PhysRevLett.117.016602

[21] S. Chi , F. Liang , H. Chen , W. Tian , H. Zhang , H. Yu , G. Wang , Z. Lin , J. Hu , H. Zhang , Adv. Mater. 2020, 32, e1904498. 10.1002/adma.201904498 31750581

[22] B. Song , C. He , S. Niu , L. Zhang , Z. Ren , X.‐J. Liu , G.‐B. Jo , Nat. Phys. 2019, 15, 911.

[23] Y. Shao , A. N. Rudenko , J. Hu , Z. Sun , Y. Zhu , S. Moon , A. J. Millis , S. Yuan , A. I. Lichtenstein , D. Smirnov , Z. Q. Mao , M. I. Katsnelson , D. N. Basov , Nat. Phys. 2020, 16, 636.

[24] B. Salmankurt , S. Duman , Philos. Mag. 2016, 97, 175.

[25] L. Guo , T.‐W. Chen , C. Chen , L. Chen , Y. Zhang , G.‐Y. Gao , J. Yang , X.‐G. Li , W.‐Y. Zhao , S. Dong , R.‐K. Zheng , ACS Appl. Electron. Mater. 2019, 1, 869.

[26] N. Clement , K. Nishiguchi , A. Fujiwara , D. Vuillaume , Nat. Commun. 2010, 1, 92. 20981020 10.1038/ncomms1092

[27] A. A. Balandin , Nat. Nanotechnol. 2013, 8, 549. 23912107 10.1038/nnano.2013.144

[28] F. D. Parmentier , L. N. Serkovic‐Loli , P. Roulleau , D. C. Glattli , Phys. Rev. Lett. 2016, 116, 227401. 27314736 10.1103/PhysRevLett.116.227401

[29] X. Cai , A. B. Sushkov , R. J. Suess , M. M. Jadidi , G. S. Jenkins , L. O. Nyakiti , R. L. Myers‐Ward , S. Li , J. Yan , D. K. Gaskill , T. E. Murphy , H. D. Drew , M. S. Fuhrer , Nat. Nanotechnol. 2014, 9, 814. 25194945 10.1038/nnano.2014.182

[30] J. C. Song , M. S. Rudner , C. M. Marcus , L. S. Levitov , Nano Lett. 2011, 11, 4688. 21936568 10.1021/nl202318u

[31] Nathaniel M. Gabor , Justin C. W. Song , Qiong Ma , Nityan L. Nair , Thiti Taychatanapat , Kenji Watanabe , Takashi Taniguchi , Leonid S. Levitov , P. Jarillo‐Herrer , Science 2011, 334, 648. 21979935 10.1126/science.1211384

[32] W. Guo , L. Wang , X. Chen , C. Liu , W. Tang , C. Guo , J. Wang , W. Lu , Opt. Lett. 2018, 43, 1647. 29652330 10.1364/OL.43.001647

[33] L. Vicarelli , M. S. Vitiello , D. Coquillat , A. Lombardo , A. C. Ferrari , W. Knap , M. Polini , V. Pellegrini , A. Tredicucci , Nat. Mater. 2012, 11, 865. 22961203 10.1038/nmat3417

[34] L. Viti , D. G. Purdie , A. Lombardo , A. C. Ferrari , M. S. Vitiello , Nano Lett. 2020, 20, 3169. 32301617 10.1021/acs.nanolett.9b05207

[35] G. Auton , D. B. But , J. Zhang , E. Hill , D. Coquillat , C. Consejo , P. Nouvel , W. Knap , L. Varani , F. Teppe , J. Torres , A. Song , Nano Lett. 2017, 17, 7015. 29016145 10.1021/acs.nanolett.7b03625

[36] L. Viti , J. Hu , D. Coquillat , W. Knap , A. Tredicucci , A. Politano , M. S. Vitiello , Adv. Mater. 2015, 27, 5567. 26270791 10.1002/adma.201502052

[37] L. Viti , J. Hu , D. Coquillat , A. Politano , C. Consejo , W. Knap , M. S. Vitiello , Adv. Mater. 2016, 28, 7390. 27315585 10.1002/adma.201601736

[38] L. Viti , A. Politano , K. Zhang , M. S. Vitiello , Nanoscale 2019, 11, 1995. 30644954 10.1039/c8nr09060b

[39] D. Zhuo , C. Jie , Z. H. U. Yi‐fan , Y. Jie , W. Zhong‐chang , Z. Kai , Chin. Opt. 2021, 14, 182.

[40] C. Guo , W. Guo , H. Xu , L. Zhang , G. Chen , G. D'Olimpio , C.‐N. Kuo , C. S. Lue , L. Wang , A. Politano , X. Chen , W. Lu , 2D Mater. 2020, 7, 035026.

[41] L. Viti , D. Coquillat , A. Politano , K. A. Kokh , Z. S. Aliev , M. B. Babanly , O. E. Tereshchenko , W. Knap , E. V. Chulkov , M. S. Vitiello , Nano Lett. 2016, 16, 80. 26678677 10.1021/acs.nanolett.5b02901

[42] Y. Niu , Y. Wang , W. Wu , J. Wen , Y. Cheng , M. Chen , S. Jiang , D. Wu , Z. Zhao , Opt. Mater. Express 2020, 10, 952.

[43] X. Tu , L. Kang , C. Wan , L. Xu , Q. Mao , P. Xiao , X. Jia , W. Dou , J. Chen , P. Wu , Opt. Express 2015, 23, 13794. 26072751 10.1364/OE.23.013794

[44] Y. Li , Y. Zhang , T. Li , X. Tang , M. Li , Z. Chen , Q. Li , Q. Sheng , W. Shi , J. Yao , J. Mater. Chem. C 2020, 8, 12148.

