# Supplementary material for: Ultrasensitive and Self‐Powered Terahertz Detection Driven by Nodal‐Line Dirac Fermions and Van der Waals Architecture
Source: Adv Sci (Weinh). 2021 Oct 19;8(23):2102088. doi: 10.1002/advs.202102088 (PMC8655208; doi:10.1002/advs.202102088)
Supplement: Supplementary file 1 — Supporting Information [file ADVS-8-2102088-s001.pdf]

ADVANCED SCIENCE
Open Access

Supporting Information

for *Adv. Sci.*, DOI: 10.1002/advs.202102088

Ultrasensitive, Broadband and Self-Powered Terahertz Detection Driven by Nodal-line Dirac Fermions and Van Der Waals Architecture

Libo Zhang, Zhuo Dong, Lin Wang, Yibin Hu, Cheng Guo, Lei Guo, Yulu Chen, Li Han, Kaixuan Zhang, Shijian Tian, Chenyu Yao, Zhiqingzi Chen, Miao Cai, Mengjie Jiang, Huaizhong Xing*, Xianbin Yu, Xiaoshuang Chen, Kai Zhang*, and Wei Lu*

Supporting information

Ultrasensitive, Broadband and Self-Powered Terahertz Detection Driven by Nodal-line Dirac Fermions and Van Der Waals Architecture

Libo Zhang, Zhuo Dong, Lin Wang*, Yibin Hu, Cheng Guo, Lei Guo, Yulu Chen, Li Han, Kaixuan Zhang, Shijian Tian, Chenyu Yao, Zhiqingzi Chen, Miao Cai, Mengjie Jiang, Huaizhong Xing*, Xianbin Yu, Xiaoshuang Chen, Kai Zhang*, and Wei Lu

Dr. L. B. Zhang, Prof. L. Wang, Dr. L. Han, Dr. K. X. Zhang, Dr. S. J. Tian, Dr. M. J. Jiang, Prof. H. Z. Xing
Department of Optoelectronic Science and Engineering, State Key Laboratory for Modification of Chemical Fibers and Polymer Materials,
Donghua University
Shanghai 201620, China

E-mail: *wanglin@mail.sitp.ac.cn*; *xinghz@dhu.edu.cn*

Dr. L. B. Zhang, Prof. Y. B. Hu, Dr. C. Y. Yao, Dr. Z. Q. Z. Chen, Dr. M. Cai, Prof. L. Wang, Prof. X. S. Chen, Prof. W. Lu
State Key Laboratory for Infrared Physics
Shanghai Institute of Technical Physics
Chinese Academy of Sciences
500 Yu-tian Road, Shanghai 200083, China
Dr. C. Guo, Prof. X. B. Yu
Research Center for Intelligent Network, Zhejiang Lab, Hangzhou, 311121, China.
Dr. Y. L. Chen
The 50th Research Institute of China Electronics Technology Group, Shanghai, 200331 China
Dr. Z. Dong, Prof. K. Zhang
CAS Key Laboratory of Nanophotonic Materials and Devices & Key Laboratory of Nanodevices and Applications, i-Lab
Suzhou Institute of Nano-Tech and Nano-Bionics (SINANO)
Chinese Academy of Sciences
Ruoshui Road 398, Suzhou, Jiangsu 215123, China
E-mail: *kzhang2015@sinano.ac.cn*
Prof. X. S. Chen, Prof. W. Lu
School of Physical Science and Technology
ShanghaiTech University
Shanghai 201210, China
Dr. Z. Dong

School of Nano-Tech and Nano-Bionics, University of Science and Technology of China.
Jinzhai Road 96, Hefei, Anhui 230026, China
Dr. L. Guo
School of Physics, Southeast University,
Nanjing 211189, China

Section 1   ZrGeSe crystal characterization

X-ray diffraction (XRD) results prove the phase purity of ZrGeSe and reflect the high crystalline quality of the single crystal. The sharp and needle-like diffraction peaks are indexed to (00l) (l=1-6) planes in Figure S1c, and the inset shows the optical microscopy image of millimeter-scale bulk ZrGeSe. Energy dispersion spectroscopy (EDS) analysis has also been carried out to validate that the material is composed of Zr, Ge, and Se elements (Figure S1d), and the atomic ratios of Zr: Ge: Se is around 32.62%: 33.95%: 33.43%, which is close to the chemical stoichiometry of ZrGeSe. A typical high-resolution transmission electron microscopy (HRTEM) image in Figure S1e demonstrates the highly ordered crystalline nature of ZrGeSe. The two lattice planes spacing along different directions are ~ 0.22 nm ~ 0.23 nm, respectively.

Section 2   The THz s-SNOM results of the ZrGeSe

To elucidate the spectral characteristics of nodal-line Dirac fermions, terahertz time-domain spectroscopy (TDS)-based scattering-type scanning near-field optical microscope (THz s-SNOM) system is utilized to delineate the interaction with incoming THz photons in Figure S2 (Supporting Information). Figure S2b and S2c simultaneously display the derived AFM topography and THz near-field nano-imaging of exfoliated ZrGeSe flake, respectively. The scattered near-field signals are high-order demodulated by a lock-in amplifier at the *n*th harmonic of the tip-tapping frequency, where the background signals are nonlinearly modulated by the distance between the tip of the cantilever and samples. As depicted in Figure S2a, there is a sizeable optical contrast between the ZrGeSe flake (marked as 6-10) and the

SiO2 (marked as 1-4) over the entire frequency range attributable to the superior electromagnetic absorption of Dirac fermions.

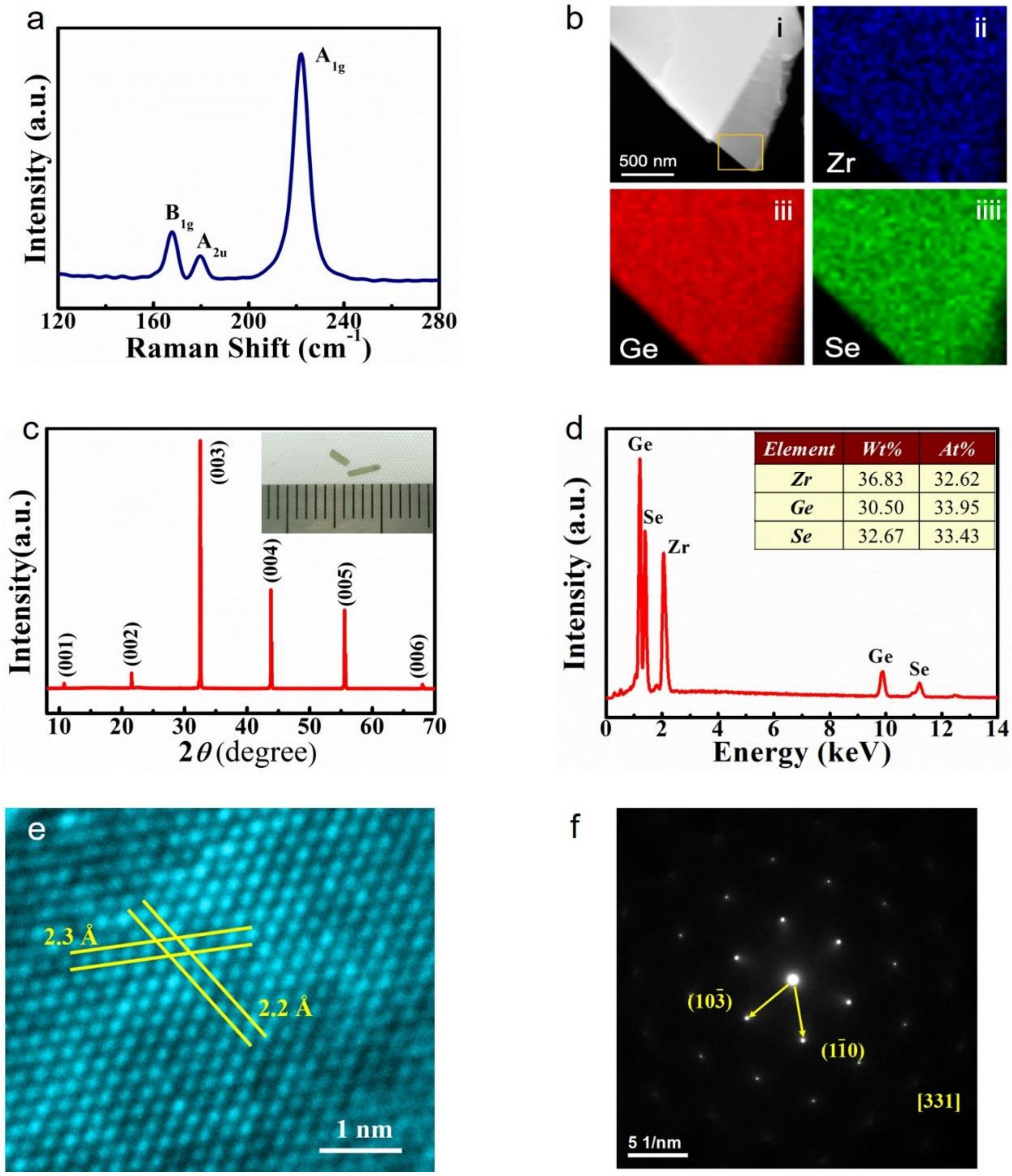

Figure S1. a. Raman spectra of the ZrGeSe on SiO_2/Si substrates. b. The TEM mapping of Zr, Ge, and Se elements. The scale bar is 500 nm. c. XRD patterns. Inset: optical microscopy of as-grown of the ZrGeSe. d. X-ray energy dispersion spectroscopy of ZrGeSe flake. Inset: corresponding element atom and weight percentage. e. HRTEM image of the single crystal. Scale bar: 1 nm. f. electron diffraction pattern. Scale bar: 5 1/nm.

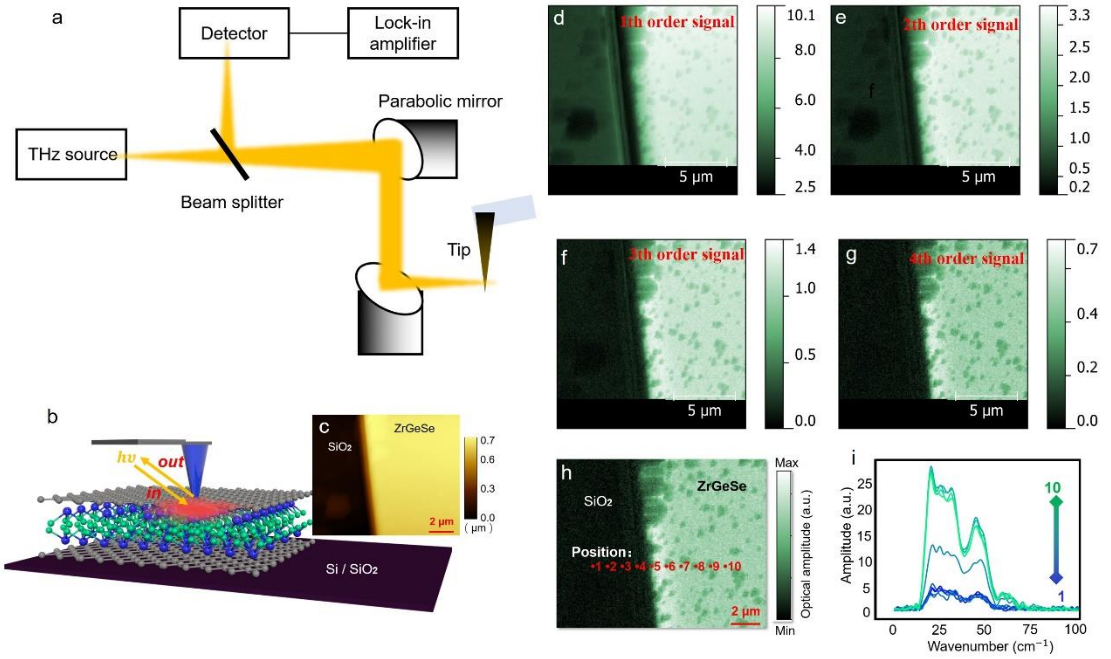

Figure S2. a. Schematic diagram of the THz s-SNOM experimental setup. The system components including a VDI multiplier, microwave source, two parabolic mirrors, beam splitter, detector lock-in amplifier, and an AFM tip. b. A THz source by VDI multiplier coupled to the AFM tip of an s-SNOM. c AFM topography and THz near-field microscopy (4th order signal) images mapping of ZrGeSe on SiO_2, respectively. d-h. THz near-field mapping of graphene on SiO_2 / Si substrate (1-4th order demodulation signals). i. Normalized THz near-field signal spectrum of the ZrGeSe sample at different positions. The region of bare SiO_2 and ZrGeSe are marked as 1-4 and 6-10, respectively, and the junction of the two is marked as 5.

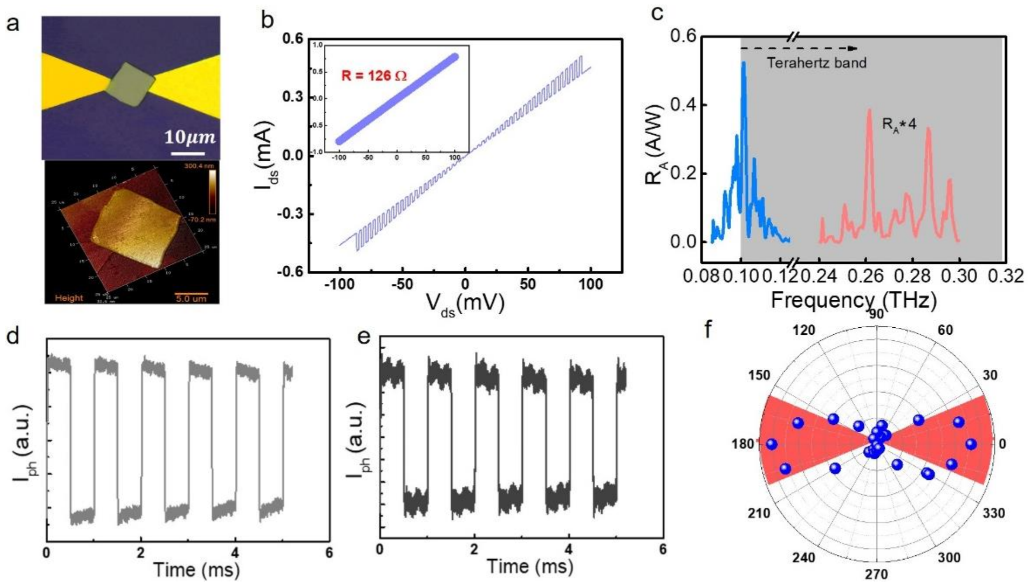

Figure S3. a. Optical microscopy and Atomic force microscopy (AFM) images of the ZrGeSe-based device with dissimilar contact metals through two lithography and electron beam evaporation processes. b. The *I*-*V* curve of the ZrGeSe-based device under the ON/OFF modulated illumination of low-energy photons with a square wave at 1 Hz. Inset: *I*-*V* curve in the dark. c. Photo-signal characteristics as a function of THz frequency (fan-shaped antenna axis parallel to the radiation polarization). d-f. Time-dependent photoresponse under the different bias voltages. f. Polar plot of the detected signal as a function of polarization angle.

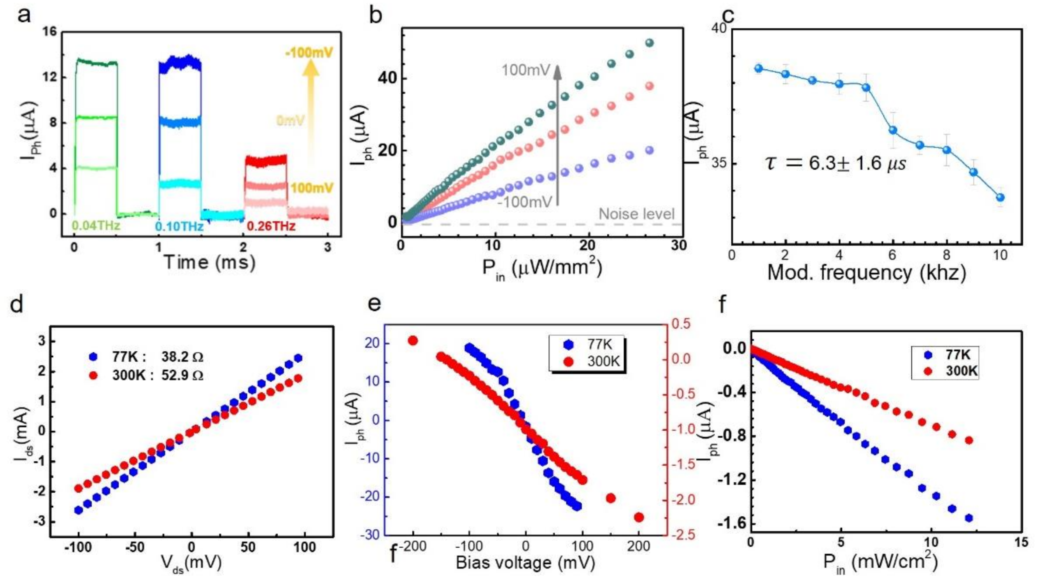

Figure S4. a. Time-dependent photoresponse under different photon frequencies (0.04 THz, 0.10 THz, and 0.26 THz). b. A sizeable dynamic regime. The photocurrent grows linearly with the incident power density (linear power dependence over more than 30 times). As a significant characteristic of a perfect THz detector, the linear dynamic regime is caused by the less internal temperature change of the THz detector system than ambient temperature. c. Photocurrent versus modulation frequency of the electromagnetic wave. d-f. Device performance characterization at low temperature. Compared with room temperature terahertz detection, the performance of the device at low temperatures has a noticeable improvement.

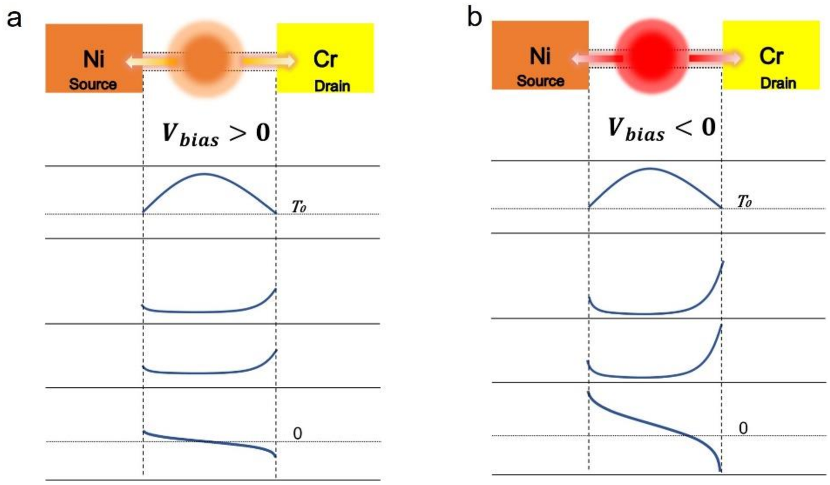

Figure S4. The Ni-Cr electrodes with a positive bias voltage a. and Ni-Cr electrodes with a negative bias voltage b. It is worth mentioning that a negative bias applied source and drain (Ni-Cr) will enlarge the Seebeck coefficient difference (ΔS), i.e., the bias-induced asymmetry, so that photocurrent has the linear growth.

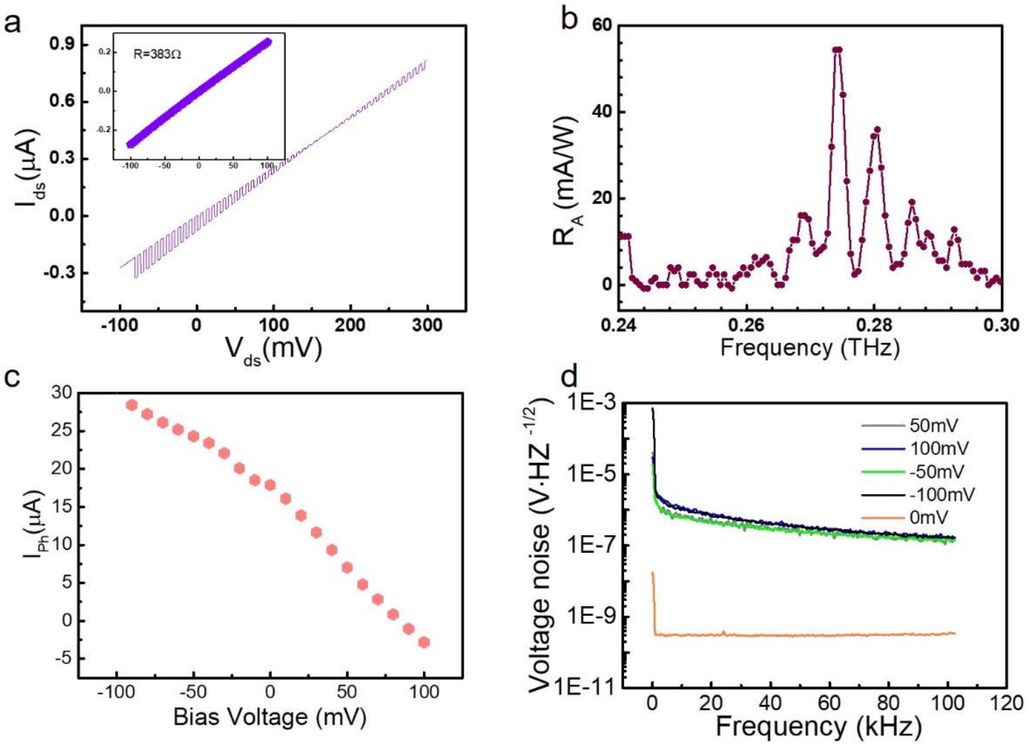

Figure S5. a. The I-V curve of the ZrGeSe-graphene device under the ON/OFF modulated illumination of low-energy photons with a square wave at 1 Hz. Inset: I-V curve in the dark. b. Frequency-dependent responsivity at room temperature. c. Photo-signal of the ZrGeSe-graphene device as a function of the bias voltage. d. Voltage noise spectra of the ZrGeSe-graphene device under the different external bias.

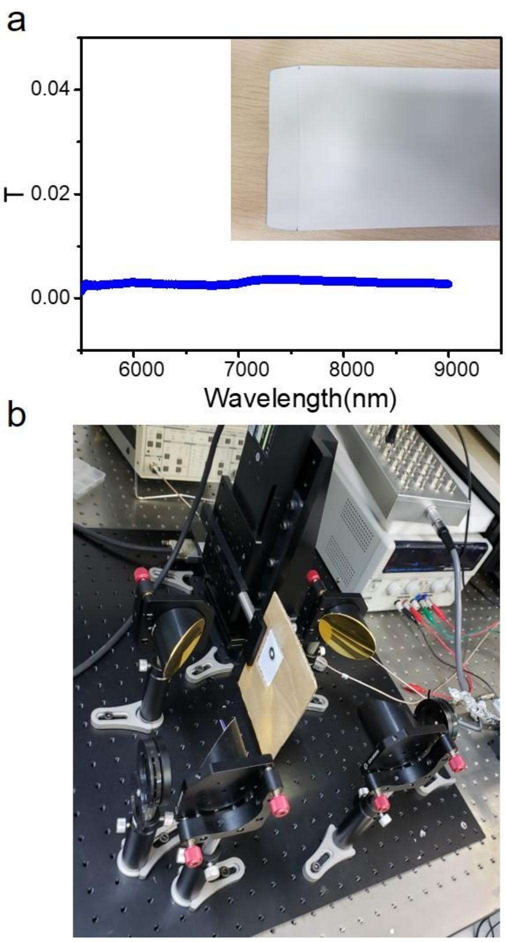

Figure S6. Real picture of Terahertz imaging. The imaging principle is that divergent terahertz light is focused by the off-axis parabolic mirror, and the optical object and photodetector are placed at the two focal points F_1, F_2, respectively.
